# Time for micro-RNAs in steatotic liver disease: a case–control study

**DOI:** 10.3389/fendo.2024.1349524

**Published:** 2024-03-14

**Authors:** Victor Constantin Stoica, Dimitri Apostol, Mihai Mircea Diculescu, Iuliana Petronela Gârdan, Daniel Adrian Gârdan, Ion Mărunțelu, Ileana Constantinescu

**Affiliations:** ^1^ Department of Gastroenterology, Fundeni Clinical Institute, Bucharest, Romania; ^2^ Immunology and Transplant Immunology, “Carol Davila” University of Medicine and Pharmacy, Bucharest, Romania; ^3^ Department of Gastroenterology, “Carol Davila” University of Medicine and Pharmacy”, Bucharest, Romania; ^4^ Department of Psychology and Education Sciences, Spiru Haret University, Bucharest, Romania; ^5^ Department of Economic Sciences, Spiru Haret University, Bucharest, Romania; ^6^ Centre for Immunogenetics and Virology, Fundeni Clinical Institute, Bucharest, Romania

**Keywords:** micro-RNA, NAFLD, MAFLD, fatty liver disease, case-control study

## Abstract

One of the challenges of modern-day living is to resist the temptation of overfeeding and sedentariness and maintain a healthy body and mind. On a favorable genetic and epigenetic background, a high-fat diet combined with lack of physical exercise constitutes the foundation for severe metabolic disturbances including steatotic liver disease. In our case–control study, we had the aim of establishing the role of selected micro-RNAs—miR-122, miR-192, miR-33a, and miR-33b—as superior biomarkers for the diagnosis and prognosis of steatotic liver in a 36-patient cohort compared to 12 healthy controls. Initial results confirmed the decline in miR-122 expression as fatty liver is progressing. However, combinations of ΔmiRs, such as ΔmiR33a_192, ΔmiR33a_122, and ΔmiR33b_122, correlate with ultrasound steatosis grade (*R* ^2^ = 0.78) while others such as ΔmiR33b_122 provide a high specificity and sensitivity in fatty liver disease with an area under the curve (AUC) of 0.85. Compared to classical biomarkers, micro-RNAs can be used for both diagnostic and prognostic purposes as their diminished expression in severe cases of steatosis is associated with higher risk of emerging hepatocellular carcinoma. Manipulating micro-RNAs through agomirs or antagomirs can be the answer to the yet unsolved problem of efficient therapy in MAFLD.

## 1 Introduction

Navigating the complexities of contemporary living poses the ongoing challenge of resisting the allure of overindulgence and sedentary habits, all while striving to cultivate a harmonious balance for a sound body and mind. Moreover, investigations into various etiologies for acute non-HepA–E hepatitis, including environmental agents, toxins, or foodborne causes, could have indirect connections to severe metabolic disturbances like steatotic liver disease (1). Against a backdrop of favorable genetics and epigenetic factors, the combination of a high-fat diet and a sedentary lifestyle lays the groundwork for profound metabolic disruptions, notably culminating in the onset of steatotic liver disease.

The above eating habits and lifestyles are also influenced by the access to medical information available for the general public but, at the same time, in a certain extent by the influence of different social groups that patients can come in contact with (2).

In the world of hepatology, the term “non-alcoholic liver disease” is losing its momentum. The concept of NAFLD is restrictive as in many cases there is even a small alcohol consumption, and it was demonstrated that there is no safe limit of alcohol use (3). Even the presence of the word “alcohol” as in NAFLD can be harmful for patients with a high risk of active stigmatization.

As the disease progresses, there are almost no organs to be spared: heart and vessels, kidneys, digestive tract, brain, etc. In this context, we will use in our article the term “metabolic associated fatty liver disease” (MAFLD) as being more comprehensive and accurate regarding the whole spectrum of comorbidities. Diagnostic criteria for MAFLD are “positive” ones, based on imaging, blood biomarkers, or histology with the presence of one out of three conditions: overweight or obesity, type 2 diabetes mellitus, or evidence of certain metabolic abnormalities (dyslipidemia, arterial hypertension, prediabetes, and increased C-reactive protein level) (3).

A relatively recent clinical trial on 765 Japanese patients argues that the MAFLD definition is more suitable than the NAFLD definition to identify patients with significant fibrosis and shows that even small amounts of alcohol intake in MAFLD patients is associated with an increase in the prevalence of significant fibrosis (4).

The disease affects at least 30% of the European population with a global prevalence estimate between 20% and 40% and up to one-fourth of affected individuals having steatohepatitis, hence a higher propensity towards liver cirrhosis or hepatocellular carcinoma (5).

MAFLD is characterized by gradual accumulation of lipids (>5% of hepatocytes in the absence of other pathogenic factors such as viral infections, drugs, and excessive alcohol intake), progressive liver inflammation, and fibrosis (6).

Increased intrahepatic fat is not inert; it is caused by insulin resistance, obesity, and systemic inflammation. Impaired free fatty acid handling determines the generation of reactive oxygen species and toxic lipid metabolites such as diacylglycerols or ceramides, causing endoplasmic reticulum and oxidative stress with the activation of unfolded protein response. Excessive inflammatory cytokine production together with the accumulation of many other periportal inflammatory cells paves the way towards fibrosis and hepatocellular carcinoma (7).

The genetic background is important in the pathogenesis of MAFLD or steatohepatitis. Examples of genes involved include PNPLA3 (patatin-like phospholipase domain containing 3), TM6SF2 (transmembrane 6 superfamily member 2), or HSD17B13 (17-β hydroxysteroid dehydrogenase 13). However, equally important or even more important are the epigenetic alterations that are reversible and heritable. Changing gene expression is possible through DNA methylation, histone acetylation, or micro-RNAs (8).

Micro-RNAs are small (18–22 nucleotides), non-coding RNAs possessing the unique feature of cleaving mRNA and silence translation. Their genesis comprises several steps: transcription of primitive miRNA from appropriate genes, cutting of pri-miRNA by RNase III Drosha and DiGeorge syndrome critical region 8 (DGCR8), export of the resulting pre-miRNA in the cytoplasm by Exportin 5, removal of the loop region by Dicer RNase III, and, after discarding one strand, the formation of a mature miRNA capable of inhibiting translation by binding to the untranslated regions of several target mRNAs known as MREs (miRNA response elements) (9).

One of the most abundant miRNAs in the liver is miR-122, representing 52% of total hepatic miRNAs. It is a key regulator of lipid metabolism, promoting lipogenesis through targeting Sirt1. Together with miR-192, it is correlated with MAFLD severity (10).

By 2015, a case–control study demonstrated an increased expression for miR-122 in NASH compared to controls (7.2-fold higher) and greater predictive ability for miR-122 for liver fibrosis compared to AST, ALT, or cytokeratin-18 (10). In other trials, hepatic miR-122 level “may be expected” to decrease as liver fibrosis worsens due to the replacement of hepatocytes with extracellular matrix (11). Also significant was the overexpression of miR-34a in MAFLD, and it was suggested that miR-34a silencing could restore Sirtuin-1 and peroxisome proliferator-activated receptor alpha (PPARα) serum levels reactivating important metabolic sensors. At that time, it was noticed that another miRNA-33a/b is involved in lipid metabolism, and its inhibition could improve fatty acid oxidation and insulin signaling (12).

Experimental trials on miR-33a and miR-33b are hindered by the fact that mice do not possess miR-33b; from here, there is a need for the creation of KI mice. Even so, genetic ablation of miR-33 in mice led to the expansion of adipose tissue depots. During obesity, miR-33a and miR-33b are upregulated in metabolically healthy individuals and miR-33a is considered as a predictor of liver inflammation and steatosis after liver transplantation (13).

However, hepatic deletion of miR-122 in mice dramatically increases the production of proteins that promote inflammation and tumor growth. Similar findings are reported regarding the activity of miR-33b, expressed from an intron located inside sterol regulatory element-binding transcription factor 1 (SREBF-1), with anti-miR-33b oligonucleotide decreasing hepatic cholesterol and triglyceride accumulation in mice liver (7). From here comes the simplistic, mechanistic idea of blocking such miRNAs, for example, miR-33b, hoping to improve liver steatosis. However, we should notice that miR-33b targets and suppresses the expression of at least 27 genes regulating tumor growth, cell cycle, proliferation, or epithelial–mesenchymal transition (EMT) (14).

## 2 Literature review and hypotheses development

For several decades, we based many of our clinical decisions on classical biomarkers of liver injury with low sensibility and specificity. Animal trials all showed important deregulation of specific miRNAs in rats fed with high-fat or high-fructose diets (15, 16).

Classical biomarkers such as ALT have an activity 3,000-fold higher in liver than in serum, whereas miR-122 concentrations are several-thousand-fold higher in liver than in other tissues. Therefore, miR-122 concentration in serum samples could be a more sensitive biomarker for hepatic injury (especially hepatotoxicity) than ALT (17).

Taking into account all of the above, we can issue our first hypothesis that serum concentrations for miR-122, -192, -33a, and -33b are correlated with ultrasound steatosis grade. Especially in the case of miR-122, such correlations have been demonstrated in both animal and human trials and in a plethora of conflicting results, a situation that motivates our research regarding the expression of miR-122 in severe fatty liver cases.

Within the scientific literature, we can find previous studies that show a possible relationship between serum concentrations for miR-122, -192, -33a, and -33b and the degree of steatosis. Thus, in a research made on 71 German subjects, 45 Italian subjects, and 31 Slovenian subjects, it can be seen that miR-122 was positively correlated with ALT and AST as well as with CK18 concentrations. MiR-122 levels were higher in children with NAFLD compared with healthy controls (18).

In another research setting, the authors have demonstrated that there was a significant increase in levels of micro-RNA-122 in obese patients with MAFLD compared to controls (*p*< 0.001). Micro-RNA-122 level was lower in patients with mild liver steatosis than in patients with moderate or severe steatosis (*p*< 0.001). It was lower in patients with a mild degree of fibrosis than in those with mild or moderate fibrosis (*p*< 0.001). Micro-RNA-122 was significantly positively correlated with low-density cholesterol and triglycerides level, and with liver enzymes, and negatively correlated with high-density cholesterol (*p*< 0.001) (19).

Therefore, our second hypothesis refers to the existence of any correlation between ultrasound steatosis grade and serum levels for miR-33a and especially miR-33b as these micro-RNAs are implicated in the regulation of cellular cholesterol and fatty acid metabolism, and recent experimental data suggest an important role for them in adipose tissue proliferation and differentiation and whole body energy expenditure (20–23).

## 3 Materials and methods

### 3.1 Research design and patient recruitment

Taking account of the previous defined hypotheses, we aimed to define the diagnostic and prognostic value for the abovementioned micro-RNA species in MAFLD, by testing several miRNA combinations for sensitivity, specificity, and area under the curve (AUC).

This is why we designed our case–control study with the aim of finding valuable new diagnostic and prognostic markers in MAFLD by using a mix of miR-122, miR-192, miR-33a, and miR-33b in patients with ultrasound diagnosed fatty liver, with special attention to miR-33b. The selection of the patients and sample collection was made starting from 2020 until 2021. A total of 36 patients from Department II of Gastroenterology within the Fundeni Clinical Institute were included in our trial according to the following criteria: patients older than 18 years, those with the ability to understand and sign the informed consent, those diagnosed with a fatty liver on ultrasound examination, and those with obesity and non-insulin-dependent type 2 diabetes were admitted.

Exclusion criteria were as follows: ethanol abuse, infectious hepatitis, autoimmune hepatitis, type 1 and insulin-dependent type 2 diabetes, therapy with pioglitazone or vitamin E during the last 2 months, hemochromatosis, and Wilson disease.

The controls are all healthy subjects with no fatty liver on ultrasound and with normal laboratory parameters. For all patients, we measured their weight, waist circumference, and height, and performed comprehensive laboratory assessment including glycated hemoglobin, C-reactive protein, transaminases, lipidic profile, and viral markers. The fibrosis-4 score (FIB-4) was calculated as follows: $\text{Fib}4=\;\frac{\text{Age }\left(\text{years}\right)\;\times \text{ AST }\left(\text{U}/\text{L}\right)}{\text{PLT }\left(10^{9}/\text{L}\right)\;\times \text{ ALT }\left(\text{U}/\text{L}\right)}$ .

### 3.2 Micro-RNA quantification

Blood samples for the detection of miR-122, miR-192, miR-33a, and miR-33b were obtained (two EDTA blood tubes, 7 mL each) from each steatosis patient and healthy control. The specimens were immediately centrifuged (2,000*g*, 20 min) and plasma was stored at −80°C until extraction. The estimation of the expression level for the miRNA panel was possible through the following procedures: total RNA extraction from blood (free and exosomal) using the MaxMax Total RNA Extraction Kit (Catalog no. A27828, Applied Biosystems, Thermo Fisher Scientific); in this step, a synthetic small RNA with a sequence corresponding to cel-miR-39 (Sequence: UCACCGGGUGUAAAUCAGCUUG, Catalog no. 10620310, Applied Biosystems, Thermo Fisher Scientific) was spiked-in to be used as an exogenous control for normalization (3 µL of cel-miR-39 diluted to 2.34 pg/µL), synthesis of complementary DNAs using TaqMan™ MicroRNA Reverse Transcription Kit (Catalog no. 4366597, Applied Biosystems, Thermo Fisher Scientific), and specific primers (Catalog no. 4427975, Applied Biosystems, Thermo Fisher Scientific). Finally, they were amplified using TaqMan™ Universal Master Mix II, no UNG (Catalog no. 4440048, Applied Biosystems, Thermo Fisher Scientific) in qRT-PCR to measure the expression level of the miRNAs.

### 3.3 Statistical analysis

Direct analysis of the level of expression between the control and steatosis groups was done through the comparative 2^−ΔΔCt^ where ΔΔCt is the difference between the ΔCt_NAFLD_ and ΔCt_control_ method.

For all continuous variables, we calculated the means and standard error values. Statistical comparisons between the healthy and steatosis groups were performed using independent-samples *t*-test and for comparison between all levels of steatosis using one-way analysis of variance (ANOVA).

Ultrasound examination was performed using Siemens ACUSON X700, with the 3.5-MHz transducer, and we considered the following aspects in the classification of patients: parenchymal echogenicity, liver texture, ultrasound attenuation, and decreased visualization of the portal and hepatic veins. Based on the above characteristics, we defined healthy liver (no abnormalities), grade 1 steatosis (echogenicity slightly increased but normal visualization of the diaphragm and intrahepatic vessels), grade 2 steatosis (echogenicity moderately increased and impaired visualization of the diaphragm or intrahepatic vessels), and grade 3 steatosis (markedly increased echogenicity and poor visualization of the diaphragm or intrahepatic vessels) (24).

To define the potential role of specified miRNAs as biomarkers, receiver operator curves (ROCs) were plotted and the AUC was determined with a 95% confidence interval. The software used for statistical analysis and plotting was R version 4.3.0.

## 4 Results

Among the 36 patients with fatty liver disease, we had 14 male patients and 22 female patients, with a mean age of 52 years (most patients were in the age group of 40–60 years) and an SD (standard deviation) of 10.88 and a mean BMI of 33.49 kg/m^2^ with an SD (standard deviation) of 5.79. Dyslipidemia was present in 24 patients out of the 36 (66.7%) patients, and impaired glucose tolerance or diabetes was present in 18 out of the 36 (50%) patients.

On ultrasound examination, 12 out of 36 patients had moderate fatty liver and 12 had severe fatty liver. Fib-4 score was also calculated and the results show a mean of 1.09, with only one patient with a score >2.5.

**Figure 1:**
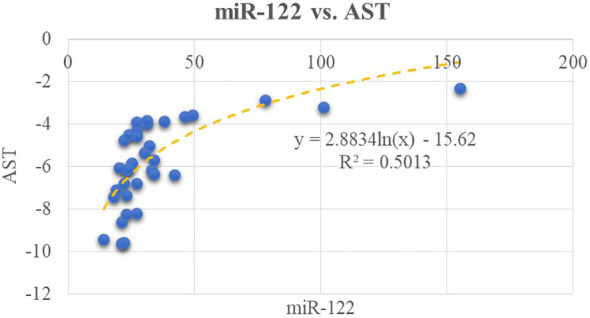
Correlations between miR-122 and AST.

**Figure 2:**
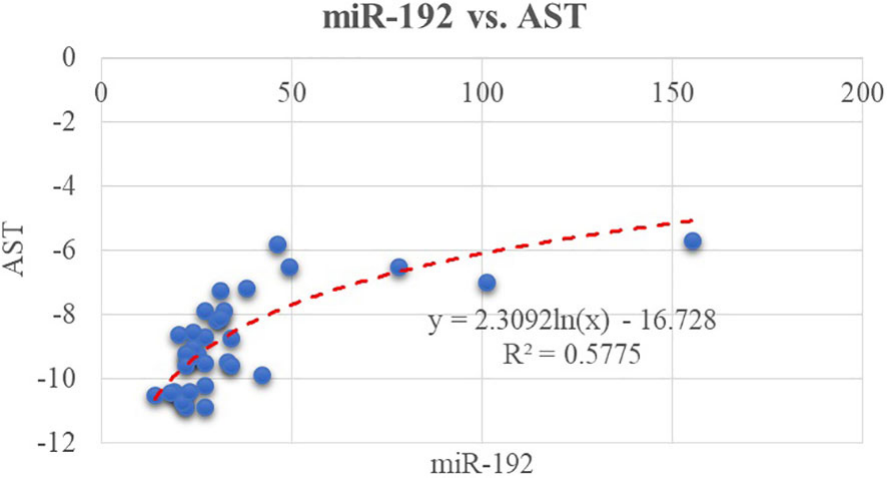
Correlation between miR-192 and another classical biomarker, AST.

We tested the possible correlations between the classical biomarkers of fatty liver (AST, ALT, and GGT) and the expression level of selected micro-RNAs. We found a moderate correlation between AST level and miR-122 (*R* ^2 =^ 0.50, *p*< 0.01); this can be viewed in **Figure 1**. In relation to ALT, there is a moderate correlation between miR-122 and ALT (*R* ^2 =^ 0.48, *p*< 0.01). A weaker correlation exists between miR-122 and GGT (*R* ^2 =^ 0.25, p = 0.002). Furthermore, miR-192 positively correlates with AST level as shown in **Figure 2**.

These results confirm our first work hypothesis, which stated the possible correlation between serum concentrations for miR-122, -192, -33a, and -33b with ultrasound steatosis grade.

The above tested correlations between potential novel biomarkers, represented by miRNAs and the classical ones, can be classified as moderate.

**Figure 3:**
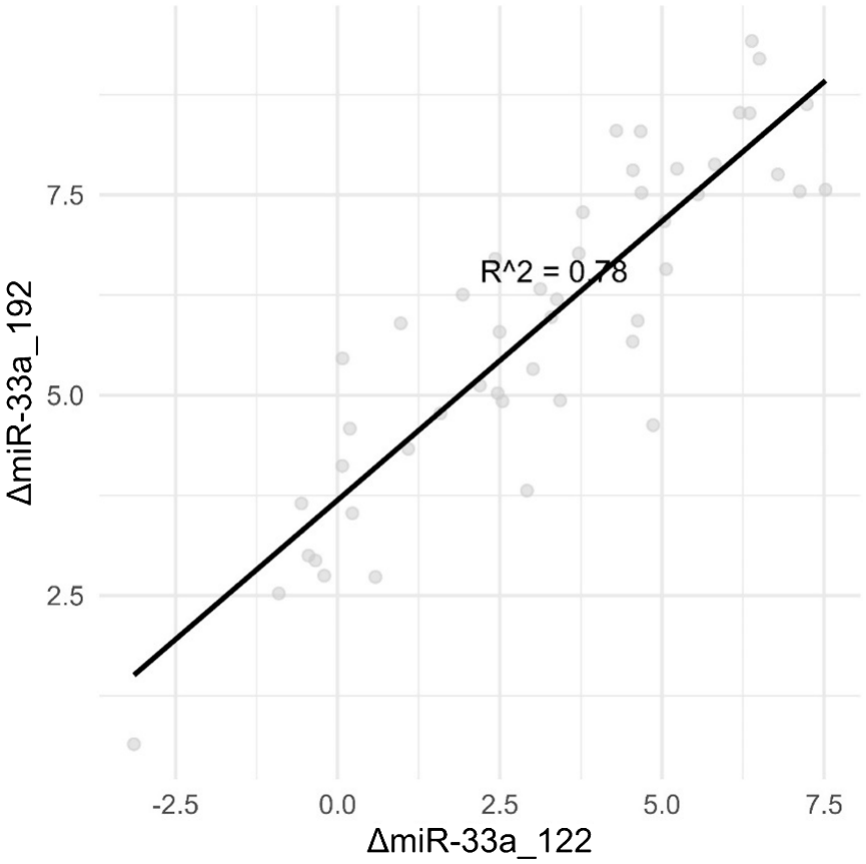
Combinations of ΔmiRNAs are strongly correlated with the degree of steatosis than single miRNAs.

As we tested several combinations of ΔmiRNAs, we discovered much stronger correlations between them, such as ΔmiR33a_192 and ΔmiR33a_122, as depicted in **Figure 3**.

The above results confirm hypothesis no. 2—the existence of a correlation between ultrasound steatosis grade and serum levels for miR-33a and especially miR-33b.

Instead of using the spiked-in cel-miR-39 as the exogenous control, directly visualizing how micro-RNAs have a combined activity is better observed by calculating the delta value between multiple combinations of micro-RNAs.

Going forward, the main advantage of using the delta value between two miRNAs is that no endogenous or exogenous (spike-in) control needs to be used while performing qPCR, thus allowing for greater repeatability between test centers. From a scientific viewpoint, the predictive ability of these miRNA pairs could be used to work backwards to the reason why these specific combinations showcase this pattern.

As we compared healthy controls with patients having grade 1 steatosis, no significant differences have been found. As the disease progresses, several combinations of tested miRNAs were identified that showed significant differences (*p*< 0.05) following the independent-samples *t*-test.

**Table 1** provides the results for the independent-samples *t*-test comparing two groups: a group of people considered “healthy” and a group of people diagnosed with a “fatty liver”. These results are presented for different miRNA-related variables. *t*-values (compared with critical values from statistical tables), *p*-values, and confidence intervals are used to assess whether there are significant differences between groups for each individual variable. Small *p*-values (* indicates values less than the 0.05 significance level and ** indicates values less than the 0.01 significance level) indicate that there is sufficient evidence to reject the null hypothesis that there are no significant differences between groups, supporting the alternative hypothesis according to which there are significant differences between the groups of “healthy” people and those with a “fatty liver”. The values in **Table 1** are suggestive of significant differences in miRNA expression for these two groups.

**Table 1:** Independent-samples *t*-test.

Variable	*t*-value	df	*p*-value	Low_95th	High_95th
ΔmiR-39_33a	2.528	37	0.016*	0.255	2.314
ΔmiR-39_33b	0.720	30	0.477	−0.464	0.971
ΔmiR-39_122	−4.333	37	0.000**	−2.697	−0.978
ΔmiR-39_192	−0.113	36	0.911	−0.668	0.598
ΔmiR-33a_33b	2.623	35	0.013*	0.233	1.830
ΔmiR-33a_122	5.419	32	0.000**	1.949	4.296
ΔmiR-33a_192	2.525	30	0.017*	0.253	2.387
ΔmiR-33b_122	5.211	35	0.000**	1.276	2.905
ΔmiR-33b_192	0.799	33	0.430	−0.445	1.022
ΔmiR-122_192	−5.637	19	0.000**	−2.473	−1.132

*p-value< 0.05; **p-value< 0.01.

**Figure 4:**
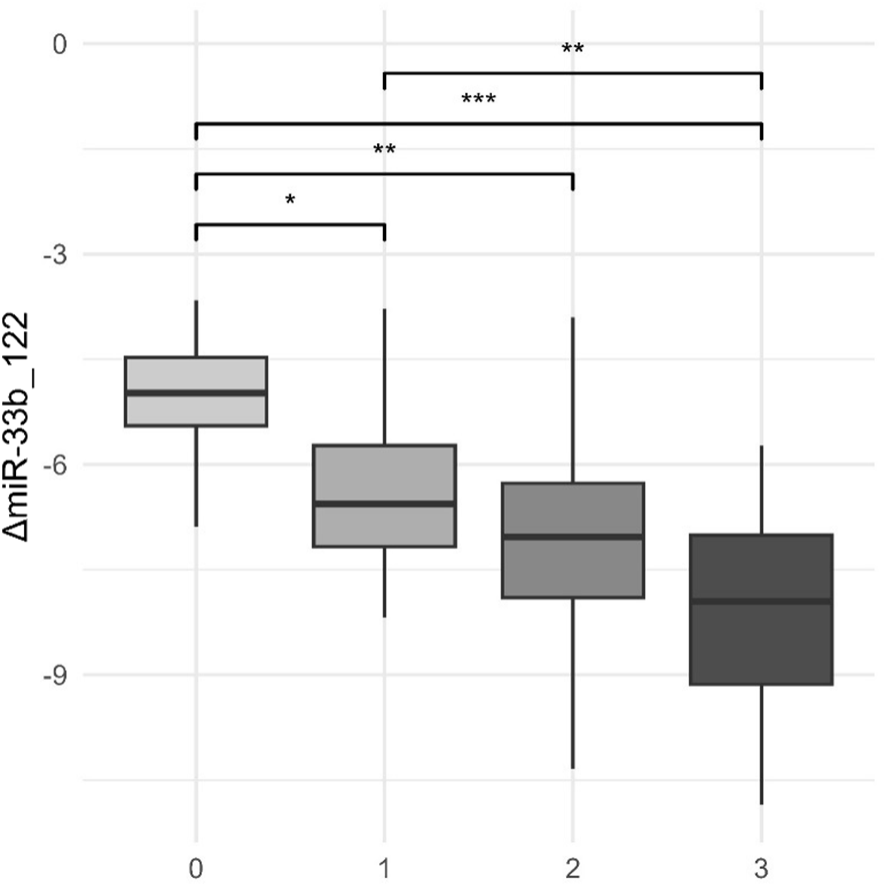
Levels of both miR-122 and miR-33b and ultrasound steatosis grade. *p-value< 0.05; **p-value< 0.01; ***p-value< 0.001.

In the given context, **Table 2** indicates that the results of ANOVA for miRNA variables according to levels of steatosis (grade 0—healthy, grade 1—mild steatosis, grade 2—moderate steatosis, and grade 3—severe steatosis) identified significant differences in the expression of these miRNAs between different grades of steatosis. The miRNA variables that showed significant differences between levels of steatosis (*p*-values lower than the usual significance levels, usually 0.05 or 0.01) are suggestive to be associated with the progression or severity of hepatic steatosis. In this case, ANOVA identified these variables as significant in relation to the degree of hepatic steatosis, indicating that the expression of specific miRNAs differed significantly between the categories of liver health assessed, from healthy individuals to patients with different degrees of steatosis (mild, moderate, and severe). This may be relevant in understanding and characterizing epigenetic changes associated with the evolution of hepatic steatosis. Among the tested combinations, we noticed ΔmiR_33a-122_, ΔmiR_33b-122_, and ΔmiR_122-192_, and one of the tested correlations is depicted in **Figure 4**.

**Table 2:** Comparison between all levels of steatosis using one-way analysis of variance (ANOVA).

Variable	*F*-value	*p*-value
ΔmiR-39_33a	22.527	0.000**
ΔmiR-39_33b	3.055	0.087
ΔmiR-39_122	9.796	0.003**
ΔmiR-39_192	0.404	0.528
ΔmiR-33a_33b	19.934	0.000**
ΔmiR-33a_122	69.995	0.000**
ΔmiR-33a_192	34.980	0.000**
ΔmiR-33b_122	27.764	0.000**
ΔmiR-33b_192	5.152	0.028*
ΔmiR-122_192	19.506	0.000**

*p-value< 0.05; **p-value< 0.01.

As liver steatosis progresses, we notice a significant fall in the expression of both miR-122 and miR-33b, here presented in combination. Grades 0–3 represent the ultrasound steatosis grade.

Regarding the possibility of using miRNAs as predictive biomarkers for fatty liver disease, we also tested several combinations, as can be seen in **Table 3**.

**Table 3:** Analysis of miRNA combinations: AUC, confidence intervals, and associated *p*-values.

Variable	AUC	Lower_95th	Upper_95th	*p*-value	Significance
ΔmiR-39_33a	0.694444444444444	0.54381233	0.84507656	0.011	*
ΔmiR-39_33b	0.599537037037037	0.43003413	0.76903995	0.250	
ΔmiR-39_122	0.805555555555556	0.68509843	0.92601268	0.000	***
ΔmiR-39_192	0.541666666666667	0.37866663	0.7046667	0.616	
ΔmiR-33a_33b	0.675925925925926	0.51866194	0.83318991	0.028	*
ΔmiR-33a_122	0.868055555555556	0.76567502	0.97043609	0.000	***
ΔmiR-33a_192	0.694444444444444	0.53746765	0.85142124	0.015	*
ΔmiR-33b_122	0.854166666666667	0.74671663	0.9616167	0.000	***
ΔmiR-33b_192	0.555555555555556	0.38144484	0.72966627	0.532	
ΔmiR-122_192	0.914351851851852	0.83551207	0.99319163	0.000	***

*p-value< 0.05; ***p-value< 0.001.

The results within **Table 3** show that several miRNA combinations have proven an AUC > 0.8, and one of them has an AUC of 0.85 (ΔmiR33b_122).

**Figure 5:**
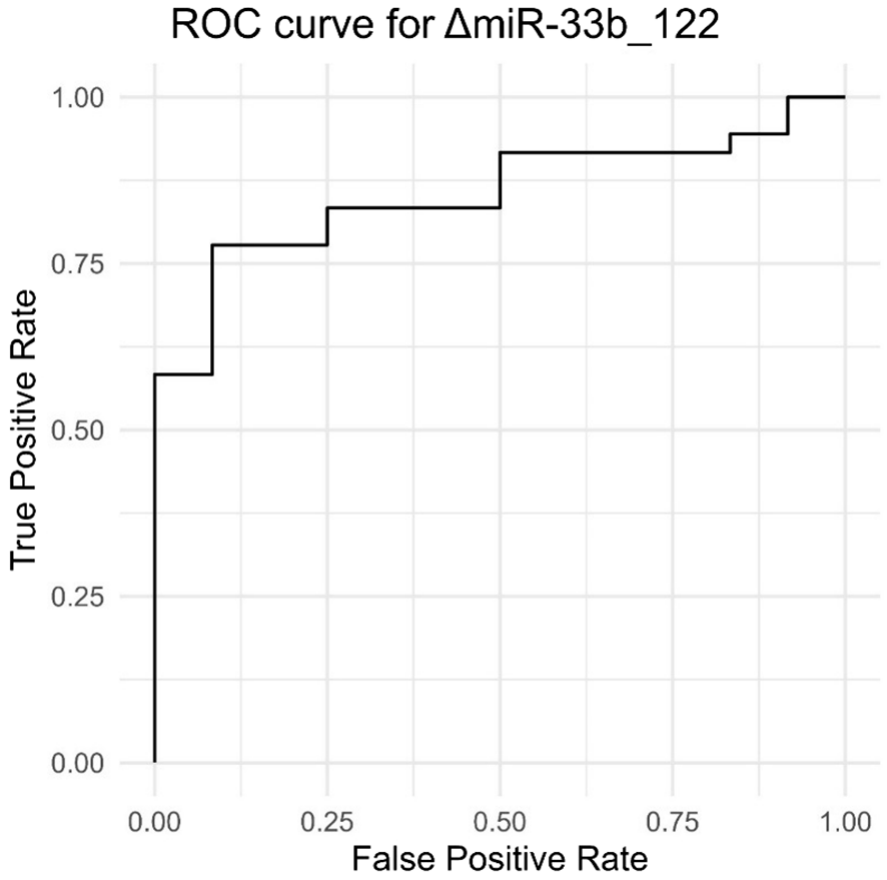
Results of true-positive rate and false-positive rate providing an ROC curve for ΔmiR33b_122.

In **Figure 5**, we can see that the ROC curve [that shows how the true-positive rate (TPR) and false-positive rate (FPR) change with the adjustment of the decision threshold] approaches the upper-left corner, thus indicating a better performance. The overall performance of the model is given by the measure of area under the curve (AUC), with higher values for AUC (closer to 1) indicating a better capacity of discrimination for the model. In our context, in which AUC is associated with ΔmiR33b_122, we can see that the ROC curve is directed upward and to the left, and the AUC is significantly greater than 0.5 (0.85), meaning that the model or test associated with ΔmiR33b_122 exhibits significantly better performance than random guessing in diagnosing or classifying the data.

Therefore, using delta values between pairs of miRNAs significantly improves their predictive abilities. Another example in which the delta value between miR-33a and miR-122 presents with an AUC of 0.819 (*p*< 0.001, 95% CI 0.689–0.949) means that we can place it well into the realm of diagnostic tests with a very high sensibility and specificity.

## 5 Discussion

A decade ago, the key role of miR-122 in terminal liver differentiation, maintaining the proper balance between hepatocyte and cholangiocyte lineages and lipid metabolism, has been clearly established. It was also noticed that anti-sense-mediated inhibition of miR-122 not only delayed liver development in zebrafish but also determined the suppression of hepatic phenotype in primary HCC with poor prognosis (25).

Conflicting results regarding miR-122 in MAFLD were available in earlier animal and human trials. From here, there were different controversial interpretations regarding its role in the pathogenesis of MAFLD (26–28).

Our study confirms a constant decrease of expression for miR-122 as the disease progresses compared to controls. We are sure that our results obtained mostly in 2022 are valid as recent trials and reviews confirm that in the later stages of liver fibrosis or before the development of liver cancer, the circulating levels of miR-122 have a downward trend (29).

A recent clinical trial published by Akuta et al. confirms our hypothesis that the decline in the expression level of circulating miR-122 in patients with MAFLD and severe fibrosis can be a strong predictor for an evolution towards HCC (30). Therefore, studying the dynamics of miR-122 expression in MAFLD could be more useful in identifying patients at high risk for HCC than transaminases or α-feto-protein.

Note that half of our patients had prediabetes or diabetes. The presence of diabetes not only augments the risk for cardiovascular mortality but also changes therapeutic decisions in MAFLD patients (31).

Induction of miR-33 expression inhibits the expression of several target genes; some are involved in reverse cholesterol transport in macrophages (ATP-binding cassette transporter ABCA1 and ABCG1), while others are implicated in fatty acid metabolism such as carnitine palmitoyltransferase 1A (CPT1A), hydroxyacyl-CoA-dehydrogenase (HADHD), and carnitine O-octanoyltransferase (CROT). By inhibiting such targets, miR-33 is responsible for the increase in the size of atheroma plaques and hepatic lipid accumulation through impaired insulin response (it represses phosphorylation of IRS-2 and AKT) (16).

In experimental trials, dual suppression of miR-33a and the NFκB pathway can attenuate atherosclerosis and hepatic steatosis (32). In addition, in mice exposed to HFD, aerobic exercise training lowers miR-33 expression and thus exerts lipid-lowering effects and induces liver autophagy with beneficial outcomes on steatosis (15).

The situation regarding miR33a and miR33b is somewhat similar. Regarding our second hypothesis, we found a decrease in the expression level for miR33a and miR33b as liver steatosis aggravates. We thought initially that it could be an adaptive mechanism as it could potentially improve liver steatosis and dyslipidemia. Other animal trials also described a decrease in the size of atheromatous plaques. However, miR33a blockage through antagomirs proved to be deleterious—mice became obese and liver steatosis did not improve (22). One of the mechanisms proposed for this phenomenon is the derepression of SREBP-1 in miR-33 KO mice (33).

However, we think that in humans, dysregulation of thermogenesis also plays an important role in the pathogenesis of fatty liver. We must not forget that humans possess two isoforms of miR-33, and miR-33a is crucial for regulating our ability to adapt to cold environments. Inhibition of miR-33a expression leads to a better peripheral thermogenesis by activating UCP (uncoupling protein 1), Dio2 type 2 deiodinase), or Prdm16 (PR domain zinc-finger-protein 16) in brown adipose tissue (21). As things cannot be so simple with miR-33s—what we get in terms of peripheral thermoregulation, we lose through the affected Thermo-genesis that is controlled by the central nervous system. This mechanism consists in the overexpression of GABRA4 and GABRB2, leading to hypothalamic inhibition of the sympathetic cold adaptive system (20). With miR33b, things were also interesting. One earlier case–control study revealed a higher miR-33b hepatic expression in morbidly obese women with NASH but no significant differences in miR-33b circulating levels no matter the liver histology (34). Our initial results suggested a marginal role for miR33b in liver steatosis. However, in combination with miR-122 or miR-192, it became a powerful tool for a sensitive diagnosis of liver steatosis (previously, we have highlighted a value of AUC of 0.85 in case of the combination between miR-122 and miR-33b).

In our study, miR-192 was found to be overexpressed according to ultrasound steatosis grade. It has a powerful effect on TGF-β production through several mechanisms: negative regulation of TGF-β E-box repressor –Zeb1/2 and targeting of Smad-interacting protein 1 (SIP1), which is another E-box repressor; another mechanism is represented by hyperglycemia (diagnosed in half of our patients). In long-standing diabetes mellitus (DM), there is an increase in TGF-β expression mediated through protein kinase C, polyol, and hexosamine pathways. MiR-192 could be the link between liver fibrosis and diabetic nephropathy (DN) as it is upregulated not only in our MAFLD patients but also in DN patients (35). The presence of T2DM could be one of the predictors of rapid fibrotic progression (36), and miR-192 might be one of the important biomarkers for disease evolution in MAFLD and DN.

To study in a more appropriate way the effects of miR-33b on the pathogenesis of NAFLD, a mouse model of miR-33b KI (knock in) was created. Mice were fed with a Gubra Amylin NASH Diet (GAN), which induces miR-33b and worsens NASH more than the classically used HFD. Mice therapy with anti-miR-33b AMO suppressed liver cholesterol and triglyceride accumulation and improved fibrosis. In addition, in mice, HSC miR-33b overexpression contributed to the exacerbation of NAFLD/NASH (37).

A recent trial confirmed that miR-33b is not just an appendix co-transcribed with miR-33a. It controls more than 27 genes that usually function as oncogenes, affecting four signaling pathways: PI3K/Akt/NF-κβ, MAPK8, Notch1, and Wnt/β-catenin (14). Mir-33b overexpression significantly prevented metformin’s decreasing effect on lipid content in HepG2 cells (38).

No matter how big the temptation of completely blocking miR-33b expression, we should withhold it for now. It is much more important to prevent the evolution towards a carcinoma (liver, colorectal, etc.). The same thing can be said for miR-33a—its anti-tumorigenic properties are extremely important; therefore, the solution that worked in animal trials was to block just the hepatic expression for miR-33a, in order to improve metabolic profile in MAFLD, but keep the cancer risk low (2)

Regarding their prognostic value, we can say that specific miRNA combinations (ΔmiRNAs in our case) could be the best solution for replacing classical biomarkers (39). It is also worth mentioning that micro-RNA serum level can be influenced by lifestyle and therapy modifications. Lifestyle interventions are now one of the therapy pillars in MAFLD. Calorie restriction (600 kcal/day) for 10 days induces remarkable changes in miRNA serum expression. For example, miR-122 is increased, while miR-19b or miR-22, miR-142, miR-143, and miR-145 levels are decreased during fasting (40). Physical exercise is another key component of lifestyle intervention in MAFLD. In mice, the aerobic endurance exercise counteracts the effect of a high-fat diet on miR-33 and miR-122 expression. This aspect should be studied in human trials as well (41).

In terms of research limitations, we may highlight the fact that patient recruitment proved to be difficult under the conditions imposed by the COVID-19 pandemic. It was hard to convince patients with a chronic condition such as MAFLD to overcome their fears and to participate in our study at Fundeni Clinical Institute. This is why we selected 36 MAFLD patients even if our initial goal was to have a higher number of patients. Healthy controls were mostly students and therefore the mean age is lower than in the case group. Some differences between the two groups might be influenced by age and less by the disease itself. In the future, we intend to recruit more patients with MAFLD, to expand the available panel of micro-RNAs, and to perform repeated (3- to 6-month intervals) ultrasound and blood tests especially for compliant patients in order to define the dynamics of micro-RNAs in the case of lifestyle modifications. Liver blockage of specific micro-RNAs should be performed with maximal caution in humans. We cannot define a dual role of MicroRNAs, but rather we can consider the existence of an extremely complex network between MIcroRNAs and their target genes. Finally, the modulation of miRNA expression may be aided by an AI algorithm, which could be an adequate response to the metabolic problems encountered in fatty liver disease without increasing the risk of neoplasia.

## 6 Conclusion

The fact that serum concentrations for miR-122, -192, -33a, and -33b are correlated with ultrasound steatosis grade confirms that there exists a clear connection between carbohydrate and lipid metabolism deregulations and poor thermoregulation, all mediated by the abovementioned micro-RNAs.

We demonstrated the existence of clear correlations between the severity of the ultrasound liver steatosis grade, and serum levels for MiR 33a abd MiR 33b in humans. Considering the previous studies carried out on laboratory mice, we can conclude that these micro RNAs described above - 33a and 33b not only influence carbohydrate and lipid metabolism but also adipose tissue proliferation and differentiation and whole body energy expenditure

Our study provides the perspective of manipulating miR-33 serum levels in order to improve central thermoregulation, resulting in a decrease in obesity and hepatic steatosis.

## Data availability statement

The original contributions presented in the study are included in the article/supplementary material. Further inquiries can be directed to the corresponding author.

## Ethics statement

The studies involving humans were approved by The Ethics Council of the Fundeni Clinical Institute, Bucharest, Romania with the approval no 55046/8.11.2019. The studies were conducted in accordance with the local legislation and institutional requirements. The participants provided their written informed consent to participate in this study.

## Author contributions

VS: Conceptualization, Investigation, Visualization, Writing – original draft. DA: Formal analysis, Investigation, Methodology, Software, Writing – review & editing. MD: Supervision, Validation, Writing – review & editing. IG: Formal analysis, Supervision, Validation, Writing – review & editing. DG: Formal analysis, Supervision, Visualization, Writing – review & editing. IM: Validation, Writing – review & editing. IC: Conceptualization, Project administration, Supervision, Writing – review & editing.
