# Corrigendum: Time for micro-RNAs in steatotic liver disease: a case–control study

**DOI:** 10.3389/fendo.2024.1463390

**Published:** 2024-07-25

**Authors:** Victor Constantin Stoica, Dimitri Apostol, Mihai Mircea Diculescu, Iuliana Petronela Gârdan, Daniel Adrian Gârdan, Ion Mărunţelu, Ileana Constantinescu

**Affiliations:** ^1^ Department of Gastroenterology, Fundeni Clinical Institute, Bucharest, Romania; ^2^ Immunology and Transplant Immunology, “Carol Davila” University of Medicine and Pharmacy, Bucharest, Romania; ^3^ Department of Gastroenterology, “Carol Davila” University of Medicine and Pharmacy”, Bucharest, Romania; ^4^ Department of Psychology and Education Sciences, Spiru Haret University, Bucharest, Romania; ^5^ Department of Economic Sciences, Spiru Haret University, Bucharest, Romania; ^6^ Center for Immunogenetics and Virology, Fundeni Clinical Institute, Bucharest, Romania

**Keywords:** micro-RNA, NAFLD, MAFLD, fatty liver disease, case-control study

In the published article, there was an error in affiliation 4. Instead of Center for Immunogenetics and Virology, Fundeni Clinical Institute, Bucharest, Romania, it should be Department of Psychology and Education Sciences, Spiru Haret University, Bucharest, Romania.

The authors apologize for this error and state that this does not change the scientific conclusions of the article in any way. The original article has been updated.

